# Frame disruptions in human mRNA transcripts, and their relationship with splicing and protein structures

**DOI:** 10.1186/1471-2164-8-371

**Published:** 2007-10-15

**Authors:** Paul Harrison, Zhan Yu

**Affiliations:** 1 Department of Biology, McGill University, Stewart Biology Building, 1205 Docteur Penfield Ave., Montreal, QC, H3A 1B1 Canada

## Abstract

**Background:**

Efforts to gather genomic evidence for the processes of gene evolution are ongoing, and are closely coupled to improved gene annotation methods. Such annotation is complicated by the occurrence of disrupted mRNAs (dmRNAs), harbouring frameshifts and premature stop codons, which can be considered indicators of decay into pseudogenes.

**Results:**

We have derived a procedure to annotate dmRNAs, and have applied it to human data. Subsequences are generated from parsing at key frame-disruption positions and are required to align significantly within any original protein homology. We find 419 high-quality human dmRNAs (3% of total). Significant dmRNA subpopulations include: zinc-finger-containing transcription factors with long disrupted exons, and antisense homologies to distal genes. We analysed the distribution of initial frame disruptions in dmRNAs with respect to positions of: (i) protein domains, (ii) alternatively-spliced exons, and (iii) regions susceptible to nonsense-mediated decay (NMD). We find significant avoidance of protein-domain disruption (indicating a selection pressure for this), and highly significant overrepresentation of disruptions in alternatively-spliced exons, and 'non-NMD' regions. We do not find any evidence for evolution of novelty in protein structures through frameshifting.

**Conclusion:**

Our results indicate largely negative selection pressures related to frame disruption during gene evolution.

## Background

Mapping transcription information (mRNAs, cDNAs, ESTs, microarray data) onto the genomic DNA is an essential part of the gene annotation process. However, many transcripts appear to have frame disruptions in them, which would interfere with the formation of a stable protein product [1]. Such frame disruptions have generally been considered symptoms of decay into a pseudogene [2,3].

Previously, we have analysed the distribution of a special case of such frame-disrupted transcripts, the 'transcribed processed pseudogene' or 'transcribed retropseudogene' [1]. Retropseudogenes are copies of messenger RNAs that have been reverse-transcribed and re-integrated into the genome, probably as a by-product of LINE-1 retrotransposition [4]. These intronless copies of genes usually decay and are deleted from the genomic DNA [1,5-8,3]. However, some retropseudogenes are transcribed, perhaps through co-option of local promoter elements, as supported by their increased density near genes [2,9].

Many mammalians genes (~40–80%) make alternatively-spliced transcripts [10]. It has previously been noted that many such alternatively spliced exons (perhaps up to ~30%) harbour premature stop codons [11,12], and thus may be considered 'pseudogenic' alternative transcripts. Other studies have demonstrated that several hundred human transcripts can be considered to harbour alternative reading frames, offset from each other by one or more frameshifts, which can be preserved for millions of years of evolution [13-15]. Frith, *et al*., found that about one-tenth of mouse cDNAs have apparent frame disruptions [16]. Sorek, *et al*., showed that ~7% of human genes generate an alternative transcript with an Alu, with the vast majority of these insertions yielding frame-disrupted mRNAs [17].

How frequent is genuine frame disruption in human mRNAs? Is it significantly associated with the positions of protein structures and exons in coding sequences? Here, to answer these and other questions, we analyze a data set of high-quality human mRNAs, using an annotation pipeline which insures that spurious frame disruptions (due simply to bad sequence alignment) are discarded. We perform statistical calculations which demonstrate non-random distribution of the initial frame disruptions in these sequences with respect to: (i) protein domain annotations, (ii) alternative exons and (iii) rules for nonsense-mediated decay (NMD). Messenger RNA transcripts that have premature stop codons greater than fifty nucleotides 5' to the last intron-exon junction of a gene are degraded by the nonsense-mediated decay (NMD) pathway [18]. Some NMD substrates have been shown to produce functional proteins in yeast and mammalian cells [19,20]. In addition, using our pipeline, we find no evidence for a role of frameshift in protein domain evolution.

## Results and discussion

### Overall statistics

**Figure 1:**
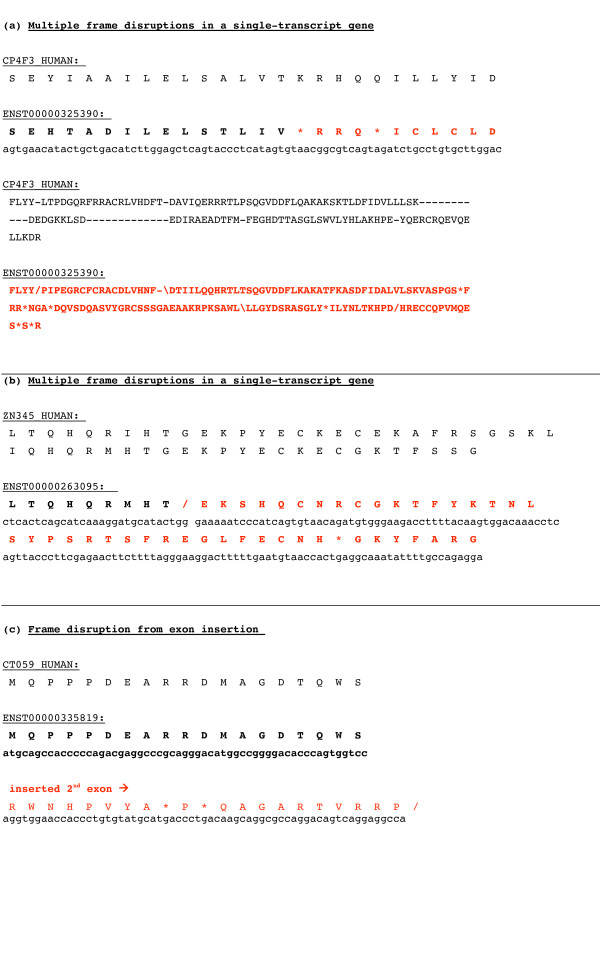
**Three examples of dmRNAs**. The translated dmRNA sequence is shown along with the corresponding nucleotide sequence; the aligning protein sequence is shown above these in each case. They are as follows: (a) a multiply-disrupted example (homologous to a cytochrome P450); (b) a multiply-disrupted example from a zinc-finger -containing transcription factor family; (c) an alternative splicing of the transmembrane sugar transporter gene, *C20orf59*, which appears to be a transmembrane sugar transporter.

Using stringent thresholds, we verified 16,153 high-quality mRNAs from the NCBI Refseq and Unigene consensus collections, through mapping onto human genomic DNA. A small subpopulation of these (419, or 3% of the total) mRNAs harbour significant frame disruptions (either frameshifts or premature stop codons) (Table 1), which is of a similar order to previous analyses of such disruptions in sets of transcripts [16,2,9]. Most of these are disrupted by frameshifts (83% of cases), rather than premature stop codons. Using a small modification to the basic annotation pipeline, we defined a small minority of these frameshifted transcripts (17, 4% of the dmRNAs) that harbour compensating frameshifts, resulting in movement back into frame. Previous analysis of mouse cDNAs also indicated that a small fraction of them (~2%) may have such compensatory frameshifts [16]. Three examples of dmRNAs are illustrated in Figure 1. There are two multiply-disrupted examples (homologous to a cytochrome P450, and to a zinc-finger -containing transcription factor), and a frameshifted alternative mRNA transcript, from the gene *C20orf59*, which appears to be a transmembrane sugar transporter.

**Table 1:** Overall statistics

**Data Set**	**Number**
Initial frame disruption is frameshift	346 (83%)
Number with compensatory frameshifts	17 (4%)
Initial frame disruption is premature stop codon	73 (17%)

**Total**	**419**

**Figure 2:**
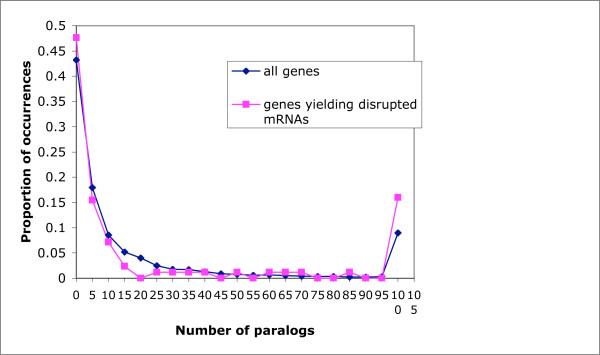
**Numbers of paralogs**. The distribution of the number of paralogs for all genes, and for genes yielding dmRNAs. The bin labeled *x *contains all values N such that *x*-5 <*N *≤ *x*.

**Figure 3:**
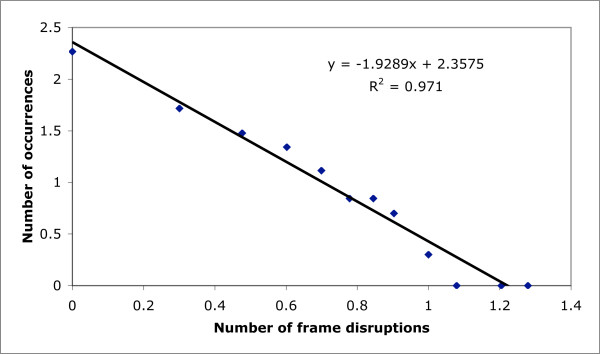
**Numbers of frame disruptions**. The number of frame disruptions in dmRNAs plotted versus the total occurrences of this number, on a log-log scale. This distribution is governed by a power law relationship, with the parameters for this linear relationship indicated on the plot.

In general, the dmRNAs demonstrate functional prevalences that are typical of the population of human transcripts in general, as judged from counting up Gene Ontology functional category annotations (Additional File 1). The duplication behaviour of the genes from which the disrupted mRNAs arise is also typical of the whole human gene complement (Figure 2; median value of 5 paralogs per gene for the disrupted mRNAs *versus *6 for the whole set; mean = 36 [± 62] *versus *32 [± 81]). However, dmRNAs have significantly fewer exons than mRNAs in general (mean = 7.9 [± 8.6] exons, compared to 10.0 [± 11.5] exons in general, P < 0.05 using normal statistics for the distribution of the sample mean). Such shorter lengths are expected from the truncating effect of frame-shifts and stop codons. A large fraction (44%) of the dmRNAs have multiple frame disruptions, with the frequencies of numbers of frame disruptions exhibiting a power-law relationship, as observed for processed pseudogenes [7,8] (Figure 3). The vast majority of frameshifts in dmRNAs (326/346, 94%)) result in truncation from premature stop codons.

We examined the etiology of the frame disruptions in dmRNAs in more detail. Some dmRNAs have apparent frame disruptions from 5' and 3' insertions of retrotransposons (24/325, 7%), or from an overlapping antisense gene (45/325, 14%). Interestingly, also, a large proportion of dmRNAs arise from antisense homologies to other distal genes (47/325, 14%). Such antisense fragments are of potential importance in transcription regulation. A functional pseudogene with antisense homology to the nitric oxide synthase gene downregulates this gene the snail *Lymnea stagnalis *[21]. These three categories of dmRNA (retroposon insertions, antisense protein homologies and overlapping gene pair homologies) comprise a subset of dmRNAs arising from 'probable UTR (untranslated region) features'. Another possible source of dmRNAs are unassigned selenoproteins [22]. We have filtered for known selenoproteins [22], but it is possible there are further cases. However, we found no indication of this, since there is no significant over-representation (relative to terminal stop codon frequencies in all Refseq mRNAs) of the opal stop codon (TGA) that is used for selenocysteine (38/57, P = 0.12, χ^2 ^test, 2 degrees of freedom).

### Exon lengths

**Figure 4:**
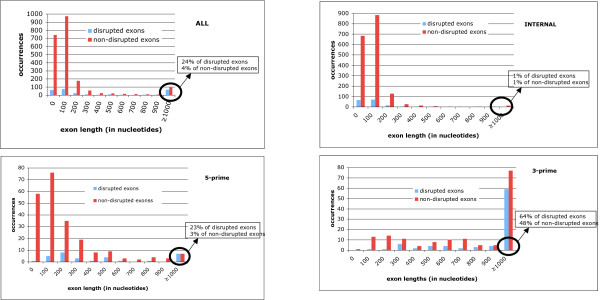
**Distribution of frame-disrupted and non-frame-disrupted exon lengths in the disrupted mRNAs**. The exon lengths are in bins labelled at either end of the bin with the upper (≤) and lower (>) bounds, with occurrences in each bin on the y axis. The percentage of exons >1000 nucleotides is given for each data set. The upper left panel is for the whole set of exons; the lower left panel for 5' exons, the upper right for internal exons, and the lower right for 3' exons.

Exons harbouring frame disruptions make up only a small fraction (288/2432, 12%) of the coding exons in their transcripts. Frame-disrupted exons are, on average, significantly longer (mean = 694 nucleotides, compared to 248 nucleotides for undisrupted exons, P < 0.001 using normal statistics for the distribution of the sample mean). Although, both frame-disrupted and non-frame-disrupted exons show a tendency for very short exon lengths (≤ 200 nucleotides), there is a greater proportion of long exons (>1000 nucleotides) in the frame-disrupted set (24% of frame-disrupted exons *versus *4% of those that are not frame-disrupted; Figure 4). To analyze exon lengths we disregarded the 'probable UTR features', but their inclusion does not change the trend observed; also, the exon length trend is maintained when exons are split into subsets of constitutive and alternatively-spliced exons. We examined the exons >1000 nucleotides in detail, and found that a significant fraction of them come from Zn-finger -containing transcription factors (36/67, 54%) with >1/3 of their sequences composed of zinc finger motifs. Zinc-finger -containing transcription factors have dynamic evolution patterns in mammals, with expansions of family sizes specific to primates and rodents [23]; large numbers of dmRNAs are a signature of other dynamically evolving mammalian gene families, such as olfactory receptors and immune system genes [1]. A significantly greater proportion of disrupted exons are at the 3' terminus of mRNAs (58/67, 87%), even if the zinc-finger -containing genes are excluded. Such 3' exons have a general tendency to be longer (51% of 3' exons in multiple-exon transcripts verified by Refseq mRNAs are ≥ 1000 nucleotides in length) (Figure 4). This greater length has been suggested to be because of a greater amount of important conserved sequences in 3' UTRs, compared to 5' ones [24].

### Positions of frame disruptions in dmRNAs

We analysed the distribution of the initial frame disruptions in the disrupted mRNAs with respect to the positioning of: (i) structural protein domains, (ii) alternatively-spliced exons, and (iii) the areas of the transcripts not susceptible to NMD (Tables 2, 3, 4, 5). In all of these analyses, we examine trends for the whole data set of dmRNAs, and the subset of these mRNAs for which the matching proteins have a *verifying alignment *in a divergent mammal or vertebrate (see *Methods *for details). The significant tendencies listed in Tables 2, 4 and 5 for the whole data set (combined stop codon and frameshift disruptions) remain significant or become more significant if those examples labelled 'probable UTR features' are removed from the data (this is illustrated for those with verifying alignments to divergent orthologs, in each case).

**Table 2:** Protein structure disruptions in mammalian messenger RNA transcripts

Type of initial frame disruption*	Alignment verification**	Protein structure disruption***		
		
		**Observed [nondisrupting : disrupting]**	**Expected [nondisrupting : disrupting]**	**Significance**
Frameshift	All cases	293 : 54	272.7 : 74.3	††
	Cases with verifying alignments	230 : 51	211.3 : 69.7	†††

Stop codon	All cases	68 : 5	55.6 : 17.4	†††
	Cases with verifying alignments	34 : 5	27.4 : 11.6	†

Frameshift *or *stop codon	All cases	360 : 59	327.5 : 91.5	†††
	Cases with verifying alignments	268 : 57	242.2 : 82.8	††
	Cases with verifying alignments (excluding probable UTR features)	174:35	153.6 : 55.4	†††

* For the last row, those with frameshifts and stop codons are pooled together.

** Verifiying alignments are significant alignments to a rodent or non-mammalian vertebrate protein, as detailed in *Methods*.

*** The ratio stands for 'the number of frame disruptions not disrupting a protein structure domain assignment versus the number that do'. A margin for ascertaining overlap with a protein domain assignment of 15 nucleotides was used in the calculations. The expectations for the statistical tests (χ^2^) are calculated by adding up the total amount of coding sequence that can be assigned to a SCOP protein structure domain for the sample of transcripts analysed in each row of the table. † stands for P < 0.05, †† for P < 0.01 and ††† for P < 0.001. The significant results remain significant to at least P < 0.05 when margins for calculating overlap with protein domains of 0, 5, 10, 20 or 25 nucleotides are also used.

**Table 3:** Distribution of initial disablements relative to zinc-finger domains *

**Margin**	**Observed [inside domain: outside domain]**	**Expected [inside domain: outside domain]**	**Significance**
0	21 : 20	26.7 : 14.3	N.S.
1	17 : 24	24.7 : 16.3	†
2	14 : 21	22.1 : 18.9	†
3	12 : 29	19.7 : 21.3	†
4	10 : 31	17.3 : 23.7	†
5	10 : 31	14.9 : 26.1	N.S.

* The format of this Table is as for Tables 2–3. The overlap margin is the number of residues that are ignored at either end of the zinc-finger domain (thus shortening the length of the defined protein motif).

**Table 4:** Frame disruption placement and alternative splicing

**Type of initial frame disruption**	**Alignment verification**	Type of exon *		
		
		**Observed [constitutive : alternative]**	**Expected [constitutive : alternative]**	**Significance**
Frameshift	All cases	191 : 156	258.8 : 88.2	†††
	Cases with verifying alignments	156 : 125	209.6 : 71.4	†††

Stop codon	All cases	33 : 40	54.4 : 18.6	†††
	Cases with verifying alignments	13 : 26	29.1 : 9.9	†††

Frameshift *or *stop codon	All cases	228 : 191	312.5 : 106.5	†††
	Cases with verifying alignments	174 : 151	242.4 : 82.7	†††
	Cases with verifying alignments (without 'probable UTR features')	114 : 95	143.8 : 65.2	†††

* Ratios are for numbers expected or observed in constitutive exons versus alternative ones. Expectations and test are performed as for Table 1.

**Table 5:** Frame disruption placement and nonsense-mediated decay

**Type of initial frame disruption**	**Alignment verification**	NMD or non-NMD region *		
		
		**Observed [NMD : non-NMD]**	**Expected [NMD : non-NMD]**	**Significance**
Frameshift	All cases	159 : 188	294.9 : 52.1	†††
	Cases with verifying alignments	122 : 159	206.6 : 74.4	†††

Stop codon	All cases	43 : 30	42.9 : 30.1	N.S.
	Cases with verifying alignments	22 : 17	17.5 : 21.5	N.S.

Frameshift *or *stop codon	All cases	201 : 218	344.5 : 74.5	†††
	Cases with verifying alignments	141 : 184	232.0 : 93.0	†††
	Cases with verifying alignments (without 'probable UTR features')	87 : 122	111.5 : 97.5	†††

* Ratios are for numbers expected or observed in NMD regions versus non-NMD ones. Expectations and tests are performed as for Table 1.

### Protein structure disruption

Do the frame disruptions in these mRNAs avoid disruption of protein structure domains? To answer this question, we analysed the distribution of initial frame disruptions in sequences relative to the placement of protein structure domains from the SCOP data (see *Methods *for details). For both frameshifts and premature stop codons, we find significant underrepresentation within protein domains (P in range *<0.05 *to *<0.001*; all of the P-values quoted for Tables 2, 3, 4, 5 are for χ^2 ^tests; see Table 2 footnote for details of statistical tests). This non-random distribution of frame disruptions is observed for a wide range of margins for definition of overlap with protein domains (between 0 and 25 nucleotides) (Table 2 footnote). This avoidance of protein structure domains is evidence for selection pressures to avoid protein structure disruption and supports a significantly negative role for frame disruption in the evolution of protein structures.

Because of the proportion of dmRNAs that contain large arrays of Zn finger domains, we also checked specifically for avoidance of disruption of Zn finger motifs. Zn finger motif assignments were taken from the feature table records of the Uniprot database [25]. We find significant avoidance of disruption of Zn finger motifs only for overlap margins of between 1 and 4 residues inclusive (Table 3).

### Alternative splicing

We examined whether there is a relationship between the position of initial frame disruptions in mRNAs and the location of alternatively spliced exons (Table 4). We find a highly significant two-fold overrepresentation of initial frame disruptions in alternatively-spliced exons (*P < 0.001*; Table 4). These correspond to almost half (~46%, 191/419) of the dmRNAs. This may arise because the selection pressure on alternative splicings that are not transcribed at high levels will be considerably less, leading to increased likelihood of frame disruption as evolution progresses [10]. It is possible that many of these frame-disrupted alternative splicings have a regulatory role [11,26]. Small numbers of the alternatively-spliced frameshifted dmRNAs arise from exon skipping (4 cases), and exon insertion (21 cases). This approximately two-fold over-representation is maintained (P < 0.05) in the subset of alternative splicings that contain SCOP [27] protein domain assignments within them.

### Transcripts not susceptible to nonsense-mediate decay

Messenger RNA transcripts that have premature stop codons greater than fifty nucleotides 5' to the last intron-exon junction of a gene are degraded by nonsense-mediated decay (NMD) [18]. We analyzed the distribution of initial frame disruptions relative to this NMD rule (Table 5). There are significantly more transcripts with frame disruptions in the 'non-NMD' region (P < 0.001; Table 5), as would be expected logically (since these transcripts would not be degraded). However, this over-representation of initial frame disruptions in the 'non-NMD' region also arises for the subsets of transcripts in which the frame disruptions disrupt a SCOP protein structure domain (P < 0.05), and which are thus unlikely to form a stable functional protein product. Such unstable protein products are more likely for shorter truncations, and thus NMD provides an evolutionary guard against excessive expression of unstable proteins [28].

### Checking for gene evolution through frame-shift formation

**Figure 5:**
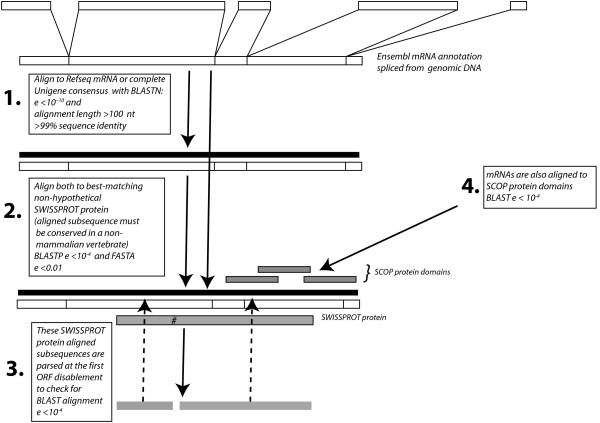
**Pipeline for annotating dmRNAs**. The steps discussed in *Methods *are illustrated schematically.

It is possible that through analysis of this comprehensive data set of dmRNAs, that we can find evidence for a *positive *role for such protein-coding frame disruptions in gene evolution. Specifically, is there evidence that such frame-shifts can produce significant structural novelties? To check this, we derived a modification for the initial pipeline (Figure 5), with matches to SCOP protein structure domains replacing those for whole protein sequences from the SWISSPROT database, finding 36 cases (9% of the dmRNAs) which produce a significant alignment for both subsequences delimited by the initial frameshift (Figure 5, step 3). However, none of these (0%) overlap another protein domain assignment in a different frame, yielding no evidence for generation of protein structure novelties through single frameshifts. Nonetheless, a more thorough analysis of multiple vertebrates would be required to provide a more conclusive perspective on the role of frameshift in protein structure evolution.

## Conclusion

We analyzed human mRNAs for both frameshift and stop codon frame disruptions, using a pipeline that was designed to discard spurious frame disruptions arising from alignment error. We performed statistical calculations and found non-random distributions of frame disruptions with respect to protein structures, alternatively-spliced exons, and 'non-NMD' regions. The significant avoidance of protein structure disruption and highly significant placement in alternatively-spliced exons (rather than constitutive ones), together with the observation of a lack of protein structure generation through frameshift, support largely negative selection pressures related to frame disruption during gene evolution.

Data from this analysis is available on request from the author.

## Methods

### Annotating disrupted mRNAs (dmRNA)

An overview of the pipeline for disrupted mRNA (dmRNA) annotation is illustrated (Figure 5).

(i) Datasets and initial alignments: Gene annotations and genome sequence data for human build 35 were obtained from the Ensembl database. Human Refseq mRNAs and Unigene consensus sequences were also obtained from the NCBI. Any 3'-end polyadenylation was removed from these NCBI transcripts before alignment. These NCBI transcripts sequences were paired with Ensembl annotations from genomic DNA, using BLASTN [29] with e-value threshold of ≤ 10^-10^, sequence identity ≥ 98% and only considering alignment lengths ≥ 100 nucleotides, that span ≥ a 0.99 fraction of each of the aligned sequences. Single-exon genes are discarded at this point.

(ii) Protein alignments to find frame disruptions: The UniProt/SwissProt protein database [25], was filtered to remove hypothetical proteins, and BLASTed against each of the Ensembl and NCBI transcript data sets, using e-value ≤ 10^-4^. The protein matches were filtered to pick the best-matching protein to each nucleotide sequence, at each position, as described previously [3]. These best-matching proteins were then masked for low-complexity sequence using the program SEG [30], and then aligned to the nucleotide sequences a second time, using FASTX/Y (e-value threshold ≤ 0.01) [31]. The extra SEG masking is necessary to remove false positives arising from a small number of repetitive protein sequences. If these second masked alignments proved significant, a final third FASTX/Y alignment was generated without masking in the sequences (with e ≤ 0.01).

Given that cDNAs may have error rates as high as as 1 in 1 × 10^-2^, and that the genomic DNA error rate is 1 × 10^-4^, we can expect that 1 in 1 × 10^-6 ^frame disruptions detected have arisen from sequencing error. Given, that we have a total of 729 frame disruptions in a total of 942,752 nucleotides, we would expect that, at most, only one of these frame disruptions has arisen from sequencing error. This implies that the data set of dmRNAs is of sufficiently high quality for in-depth bioinformatic analysis.

*Alu *elements are a common pollutant in protein databases [17]. We obtained a large number of matches to *Alu *elements producing dmRNAs; since these are not a focus of our current analysis, they were removed using protein-level translations of *Alu *sequences, with a more accommodating BLAST threshold of e ≤ 0.01.

We removed selenoproteins from the dataset, since these are a known example of a re-coding phenomenon [22]. This was achieved through protein-level BLAST comparisons (e ≤ 10^-4^) to the determined human selenoproteome, downloaded from the SelenoDB database [22].

To insure that we are not considering spurious frameshifts arising from bad protein annotations, we used an additional filter to insure that the protein reading frames in question are well conserved in a distant mammal or vertebrate. We required that the matching protein is conserved in a rodent or non-mammal vertebrate (with BLAST e-value ≥ 10^-4^) over ≥ 95% of its length.

(iii) Realignment to remove spurious frame disruptions: Spurious frame disruptions can arise as alignment errors when comparing a protein to a nucleotide sequence [3]. Such spurious frame disruptions are more frequent in more divergent aligned sequences; they are typically near the ends of aligned subsequences, and can also arise in compositionally-biased or low-complexity regions [3]. To insure that spurious frame disruptions are not considered in the present analysis, we parse the disrupted coding sequences at the initial frame disruption into two subsequences, and require that both of these subsequences align significantly to the original matching protein (BLAST e-value ≤ 10^-4^).

In addition, we checked for compensatory frameshifts (*i.e*., pairs of frameshifts that move a coding sequence out of frame, and then back into frame). It is possible that compensatory frameshifts provide a mechanism for generating sequence diversity in proteins over evolution. We checked for compensatory frameshifting, using an additional filter in the initial pipeline at step 3 (Figure 5). For every case of an initial frameshift, we checked for a second frameshift 3' to it in the transcript that corrects for the first frameshift. Then we checked whether the three subsequences delimited by these two frameshifts all align significantly with the original matching protein.

(iv) Protein domain matching: The Ensembl and NCBI transcripts were searched against the ASTRALSCOP protein domain database [27], using BLAST (e-value ≤ 10^-4^), and the best-matching domains at each position in a transcript were retained, as described previously [8,3].

Specifically, also, we extracted zinc-finger motif assignments from the feature table records of the UniProt/SWISSPROT database [25].

### Checking for evolution through frameshifting

We checked for evidence of protein structure evolution through frameshift, using a modification of the initial pipeline (Figure 5). All steps were performed as before, except with SCOP protein domain sequences in lieu of SWISSPROT sequences. Then, we checked for any significant undisrupted matches to other protein domains at the same positions as these frameshifted protein domain matches, using a similar protocol of BLAST database searching, followed by refined alignment using FASTX/Y.

## Authors' contributions

PH wrote the paper and performed most of the data analysis. ZY did some initial data analysis. Both authors have read and approved the final manuscript.

## Supplementary Material

Additional file 1 Supplementary Table 1: GO categories. The most abundant Gene Ontology (GO) functional categories for different data sets of exons are listed. Click here for file

## References

[B1] Harrison P, Gerstein M (2002). Studying genomes through the aeons: protein families, pseudogenes and proteome evolution. J Mol Biol.

[B2] Harrison PM, Zheng D, Zhang Z, Carriero N, Gerstein M (2005). Transcribed processed pseudogenes in the human genome: an intermediate form of expressed retrosequence lacking protein-coding ability. Nucleic acids research.

[B3] Harrison PM, Hegyi H, Balasubramanian S, Luscombe NM, Bertone P, Echols N, Johnson T, Gerstein M (2002). Molecular fossils in the human genome: identification and analysis of the pseudogenes in chromosomes 21 and 22. Genome Res.

[B4] Esnault C, Maestre J, Heidmann T (2000). Human LINE retrotransposons generate processed pseudogenes. Nature genetics.

[B5] Karro JE, Yan Y, Zheng D, Zhang Z, Carriero N, Cayting P, Harrison P, Gerstein M (2007). Pseudogene.org: a comprehensive database and comparison platform for pseudogene annotation. Nucleic acids research.

[B6] Yu Z, Morais D, Ivanga M, Harrison PM (2007). Analysis of the role of retrotransposition in gene evolution in vertebrates. BMC Bioinformatics.

[B7] Zhang Z, Harrison P, Liu Y, Gerstein M (2003). Millions of years of evolution preserved: a comprehensive catalog of the processed pseudogenes in the human genome. Genome Res.

[B8] Zhang Z, Harrison P, Gerstein M (2002). Identification and analysis of over 2000 ribosomal protein pseudogenes in the human genome. Genome Res.

[B9] Vinckenbosch N, Dupanloup I, Kaessmann H (2006 ). Evolutionary fate of retroposed gene copies in the human genome. Proc Natl Acad Sci USA.

[B10] Modrek B, Lee CJ (2003). Alternative splicing in the human, mouse and rat genomes is associated with an increased frequency of exon creation and/or loss. Nat Genet.

[B11] Lewis BP, Green RE, Brenner SE (2003). Evidence for the widespread coupling of alternative splicing and nonsense-mediated mRNA decay in humans. Proc Natl Acad Sci USA.

[B12] Letunic I, Copley RR, Bork P (2002). Common exon duplication in animals and its role in alternative splicing. Hum Mol Genet.

[B13] Liang H, Landweber L (2006). A genome-wide study of dual coding regions in human alternatively spliced genes. Genome Res.

[B14] Raes J, Van de Peer Y (2005). Functional divergence of proteins through frameshift mutations. Trends Genet.

[B15] Okamura K, Feuk L, Marques-Bonet T, Navarro A, Scherer SW (2006). Frequent appearance of novel protein-coding sequences by frameshift translation. Genomics.

[B16] Frith MC, Wilming LG, Forrest A, Kawaji H, Tan SL, Wahlestedt C, BAjic VB, Kai C, Kawai J, Carninci P, Hayashizaki Y, Bailey TL, Huminiecki L (2006). Pseudo-messenger RNA: phantoms of the transcriptome. PLoS Genet.

[B17] Sorek R, Ast G, Graur D (2002). Alu-containing exons are alternatively spliced. Genome Res.

[B18] Maquat LE (2004). Nonsense-mediated mRNA decay: splicing, translation and mRNP dynamics. Nat Rev Mol Cell Biol.

[B19] Ford AS, Guan Q, Neeno-Eckwall E, Culbertson MR (2006). Ebs1p, a negative regulator of gene expression controlled by the Upf proteins in the yeast Saccharomyces cerevisiae. Eukaryot Cell.

[B20] Inoue K, Lupski JR, Khajavi M, Ohyama T, Hirabayashi S, Wilson J, Reggin JD, Mancias P, Butler IJ, Wilkinson MF, Wegner M (2004). Molecular mechanism for distinct neurological phenotypes conveyed by allelic truncating mutations. Nat Genet.

[B21] Korneev SA, Park JH, O'Shea M (1999). Neuronal expression of neural nitric oxide synthase (nNOS) protein is suppressed by an antisense RNA transcribed from an NOS pseudogene. J Neurosci.

[B22] Fomenko DE, Xing W, Adair BM, Thomas DJ, Gladyshev VN (2007). High-throughput identification of catalytic redox-active cysteine residues. Science.

[B23] Hamilton AT, Huntley S, Tran-Gyamfi M, Baggott DM, Gordon L, Stubbs L (2006). Evolutionary expansion and divergence in the ZNF91 subfamily of primate-specific zinc finger genes. Genome Res.

[B24] Scofield DG, Hong X, Lynch M (2007). Position of the final intron in full-length transcripts: determined by NMD?. Mol Biol Evol.

[B25] Apweiler R, Bairoch A, Wu CH, Barker WC, Boeckmann B, Ferro S, Gasteiger E, Huang H, Lopez R, Magrane M, O'Donovan C, Redaschi N, Yeh LS (2004). UniProt: the Universal Protein knowledgebase. Nucleic acids research.

[B26] Lareau LF, Inada M, Green RE, Wengrod JC, Brenner SE (2007). Unproductive splicing of SR genes associated with highly conserved and ultraconserved DNA elements. Nature.

[B27] Chandonia JM, Hon G, Walker NS, Lo Conte L, Koehl P, Levitt M, Brenner SE (2004). The ASTRAL Compendium in 2004. Nucleic acids research.

[B28] Mendell JT, Sharif NA, Meyers J, Martinez F, Dietz HC (2004). Nonsense surveillance regulates expression of diverse classes of mammalian transcripts and mutes genomic noise. Nat Genet.

[B29] Altschul SF, Madden TL, Schaffer AA, Zhang J, Zhang Z, Miller W, Lipman DJ (1997). Gapped BLAST and PSI-BLAST: a new generation of protein database search programs. Nucleic acids research.

[B30] Wootton JC, Federhen S (1996). Analysis of compositionally biased regions in sequence databases. Methods Enzymol.

[B31] Pearson WR (2000). Flexible sequence similarity searching with the FASTA3 program package. Methods Mol Biol.

