# Supplementary material for: Frame disruptions in human mRNA transcripts, and their relationship with splicing and protein structures
Source: BMC Genomics. 2007 Oct 15;8:371. doi: 10.1186/1471-2164-8-371 (PMC2194788; doi:10.1186/1471-2164-8-371)
Supplement: Additional file 1 — Supplementary Table 1: GO categories. The most abundant Gene Ontology (GO) functional categories for different data sets of exons are listed. [file 1471-2164-8-371-S1.doc]

Supplementary Table 1: Gene Ontology functional categories for different types of exon in human *

Alternatively-spliced All exons Disabled alternatively-spliced

exons exons

# Category Number Category Number Category Number

**GO:0005634** 3970 **GO:0005634** 1817 **GO:0005634** 30

**GO:0016021** 3190 **GO:0016021** 1278 **GO:0008270** 27

**GO:0016020** 2612 **GO:0016020** 1186 **GO:0006355** 21

**GO:0005515** 2360 **GO:0005515** 1152 **GO:0016021** 20

**GO:0008270** 2069 **GO:0006355** 1012 **GO:0003676** 19

**GO:0006355** 2029 **GO:0008270** 1010 **GO:0005524** 14

**GO:0005524** 1687 **GO:0005524** 916 **GO:0016020** 12

**GO:0003677** 1339 **GO:0003677** 638 **GO:0004872** 10

**GO:0007165** 1264 **GO:0016740** 621 **GO:0003677** 10

**GO:0016740** 1263 **GO:0007165** 547 **GO:0005887** 9

**GO:0004872** 1242 **GO:0003676** 528 **GO:0005515** 9

**GO:0016787** 1171 **GO:0004872** 527 GO:0006468 8

**GO:0005887** 1108 **GO:0016787** 522 **GO:0016740** 7

**GO:0005737** 1059 **GO:0003700** 503 GO:0004674 7

**GO:0003700** 1052 **GO:0005737** 495 GO:0006629 6

**GO:0003676** 1029 **GO:0000166** 493 GO:0046872 5

**GO:0000166** 1023 **GO:0005887** 452 GO:0008372 5

GO:0046872 931 GO:0005509 436 GO:0007242 5

GO:0005554 910 GO:0005554 434 **GO:0007165** 5

GO:0007186 900 GO:0008372 420 GO:0006810 5

* Human GO annotations were compiled as previously described [ref.] The top twenty GO category

numbers are listed for each of the sets: all exons; all alternatively-spliced exons; all disabled alternatively-spliced exons. GO categories that are found in ‘alternatively-spliced exons’, and that are also found in ‘all exons’ and ‘disabled alternatively-spliced exons’, are in bold.
